# Risk prediction models for cognitive impairment in patients with cerebral small vessel disease: a systematic review and meta-analysis

**DOI:** 10.3389/fnagi.2026.1679020

**Published:** 2026-02-25

**Authors:** Ting Li, Wen Shen, Yun Wang, Ping Jia, Xia Zeng

**Affiliations:** 1 School of Nursing, Chengdu University of Traditional Chinese Medicine, Chengdu, Sichuan, China; 2 Intensive Care Medicine Center, Sichuan Academy of Medical Sciences and Sichuan Provincial People’s Hospital, Affiliated Hospital of University of Electronic Science and Technology of China, Chengdu, Sichuan, China; 3 Emergency Intensive Care Unit, Sichuan Academy of Medical Sciences and Sichuan Provincial People’s Hospital, Affiliated Hospital of University of Electronic Science and Technology of China, Chengdu, Sichuan, China

**Keywords:** cerebral small vessel disease, cognitive impairment, meta-analysis, risk prediction models, systematic review

## Abstract

**Objective:**

This study systematically evaluates the risk prediction models for cognitive impairment in patients with cerebral small vessel disease (CSVD) and explores the predictive factors for cognitive impairment to provide effective guidance for the future development of higher-quality prediction models.

**Methods:**

A computer-based search was conducted using the following databases: Wanfang Database, China National Knowledge Infrastructure (CNKI), VIP Database, China Biomedical Literature Database, EMBASE, Web of Science, PubMed, and The Cochrane Library. The search aimed to identify studies on risk prediction models for cognitive impairment in patients with CSVD, covering the period from the inception of each database up to 15 June 2025. A meta-analysis of the predictive factors and the area under the receiver operating characteristic curve (AUC) values of the models was performed using RevMan 5.4 and R software, respectively. The Prediction model Risk of Bias ASsessment Tool (PROBAST) was used for screening, data extraction, and assessment of the risk of bias in the included studies.

**Results:**

A total of 19 studies were selected for inclusion, resulting in the development of 27 risk prediction models for cognitive impairment. The AUC of all models was greater than 0.7. PROBAST assessment results indicated a high risk of bias across the studies, but the applicability of the models was relatively good. Statistical analysis using R software revealed an AUC of 0.87 (95% CI: 0.79–0.92) and 0.85 (95% CI: 0.82–0.88) for the models, indicating good predictive performance. Meta-analysis results showed that hypertension, homocysteine (Hcy), high CSVD burden, age, diabetes, and the triglyceride–glucose (TyG) index (all with *p* < 0.05) were the major predictors of cognitive impairment.

**Conclusion:**

The performance and quality of existing risk prediction models for cognitive impairment in patients with cerebral small vessel disease (CSVD) still require improvement. The majority of the models lack external validation and appropriate calibration methods, and many are retrospective studies, which increases the overall risk of bias. Future research should focus on exploring more advanced machine learning algorithms, optimizing study designs, and emphasizing external validation to enhance the generalizability of the models. This would help build more universally applicable prediction models, thereby guiding the clinical implementation of targeted preventive measures.

**Systematic review registration:**

https://www.crd.york.ac.uk/prospero/, identifier CRD420251074647.

## 1 Introduction

Cerebral small vessel disease (CSVD) is a pathological syndrome triggered by various etiologies, primarily affecting the small arteries, arterioles, capillaries, and venules within the brain. Its typical neuroimaging features include damage to the cerebral white matter and deep gray matter, usually identified through imaging biomarkers such as white matter hyperintensities, lacunar infarcts, cerebral microbleeds, and cerebral microinfarcts (Markus and de Leeuw, 2023). These changes reflect the underlying pathological mechanisms of endothelial dysfunction in the cerebral vasculature and impaired cerebral blood flow perfusion (Markus and Joutel, 2025). Such pathological damage directly disrupts the structural integrity of brain tissue and the function of neural circuits, thereby contributing to vascular cognitive impairment (VCI). Epidemiological data show (Iadecola et al., 2019) that CSVD-related cognitive impairment contributes to up to 45% of cases among patients with cognitive impairment, making it a leading cause of VCI. As the global population ages, the incidence of CSVD increases significantly with age, directly contributing to the rising burden of cognitive disorders worldwide (Mok and Kim, 2015). This burden manifests in several ways: for patients, in addition to cognitive decline, there is a higher risk of anxiety and depression, along with a significant decrease in quality of life; for families and society, the long-term care requirements and medical resource consumption associated with the disease further exacerbate the pressure on public health systems (Hussenoeder et al., 2020). However, cognitive impairment caused by CSVD is often not detected early because of the subtle onset of symptoms. Its core features include slowed information processing speed and impaired executive function, with relatively preserved short-term memory. The atypical clinical features of CSVD make its diagnosis heavily reliant on clinical experience, which can be influenced by individual factors such as education level and underlying comorbidities (Salvadori et al., 2023), thereby complicating the early detection of disease progression. If the intervention is delayed, cognitive functions such as memory, language, and visuospatial abilities may gradually deteriorate, ultimately progressing to dementia and significantly increasing the difficulty of treatment and the risk of disability. The close relationship between CSVD and stroke further underscores the importance of early intervention. Approximately 20% of strokes are caused by CSVD. There is a clear bidirectional risk interaction between the two, and stroke significantly increases the risk of dementia. Conversely, cognitive decline also raises the likelihood of stroke occurrence (Das et al., 2019). This vicious cycle underscores the importance of identifying high-risk individuals early using effective tools to break the chain of disease progression. In recent years, an increasing number of risk prediction models for cerebral small vessel disease–related cognitive impairment have been proposed. However, substantial heterogeneity exists among these models in terms of predictor selection, statistical methodologies, validation strategies, and reporting transparency. The majority of models are developed using small, single-center cohorts, lack external validation, or fail to report key performance metrics, such as calibration and discrimination, raising concerns regarding their generalizability and clinical applicability. To date, systematic reviews of cognitive impairment related to cerebral small vessel disease are limited, and existing studies have primarily focused on identifying risk factors rather than evaluating the prediction models themselves. Previous reviews have not applied standardized frameworks such as the Checklist for Critical Appraisal and Data Extraction for Systematic Reviews of Prediction Modelling Studies (CHARMS) or PROBAST to assess methodological quality, nor have they synthesized the predictive performance of available models. Furthermore, Chinese-language publications, which are a significant part of research in this field, have not been included in prior analyses, substantially limiting the applicability of existing evidence to Asian clinical populations.

Therefore, this study aims to systematically review and meta-analyze prediction models for cerebral small vessel disease–related cognitive impairment by incorporating both English- and Chinese-language studies, with the following objectives: (1) to assess the methodological quality and applicability of existing models; (2) to synthesize the predictive performance of these models; and (3) to provide evidence-based recommendations to support model optimization and clinical translation.

## 2 Materials and methods

The study protocol is registered on the International Prospective Register of Systematic Reviews (PROSPERO; CRD: 420251074647).

### 2.1 Inclusion and exclusion criteria

Inclusion criteria were as follows:

1. Patients with a clear diagnosis of CSVD, where the diagnostic criteria must align with international consensus or authoritative guidelines.
2. Study type: case–control or cohort studies.
3. The focus of this study must involve the development, validation, or updating of CSVD-related cognitive impairment risk-prediction models.
4. Language of the literature: Chinese or English.

Exclusion criteria were as follows:

1. Reviews, conference abstracts, or duplicate publications.
2. Studies in which the full text or data cannot be accessed.
3. Studies that analyzed only risk factors for CSVD-related cognitive impairment without constructing a prediction model.

### 2.2 Study search strategy

A comprehensive, computer-based search was conducted across multiple databases, including the VIP Database, China National Knowledge Infrastructure (CNKI), Wanfang Database, China Biomedical Literature Database, EMBASE, Web of Science, PubMed, and The Cochrane Library. The search covered all available records up to 15 June 2025 and utilized both subject headings and free terms combined with Boolean operators. The Boolean search strategy for PubMed is presented in Table 1.

**Table 1:** The retrieval process of PubMed.

#1	Cerebral small vessel disease [MeSH Terms]
#2	(((((((cerebral small vessel disease [Title/Abstract]) OR (CSVD [Title/Abstract])) OR (white matter lesion [Title/Abstract])) OR (cerebral micro bleeds [Title/Abstract])) OR (lacunar infarction [Title/Abstract])) OR (cerebral atrophy [Title/Abstract])) OR (white matter hyperintensity [Title/Abstract]))
#3	#1 OR #2
#4	dementia* [MeSH Terms]
#5	(((((((dementia*[Title/Abstract]) OR (Alzheimer disease [Title/Abstract])) OR (cognitive dysfunction [Title/Abstract])) OR (cognition disorder*[Title/Abstract])) OR (cognitive impairment [Title/Abstract])) OR (Cognitive decline [Title/Abstract])) OR (Dementia [Title/Abstract]))
#6	#4 OR #5
#7	(((((((((((prediction*[Title/Abstract]) OR (predict*[Title/Abstract])) OR (prediction model [Title/Abstract])) OR (risk prediction [Title/Abstract])) OR (risk assessment [Title/Abstract])) OR (risk evaluation [Title/Abstract])) OR (risk score [Title/Abstract])) OR (risk stratification model [Title/Abstract])) OR (prediction tool*[Title/Abstract])) OR (prognostic model [Title/Abstract])) OR (stratification model [Title/Abstract]))
#8	#3 AND #6 AND #7

(“CSVD” OR “cerebral small vessel disease” OR “white matter lesion” OR “cerebral microbleeds” OR “lacunar infarction” OR “cerebral atrophy” OR “white matter hyperintensity”).

AND (“dementia” OR “Alzheimer’s disease” OR “cognitive dysfunction” OR “cognition disorder” OR “cognitive impairment” OR “cognitive decline”).

AND (“prediction*” OR “predict*” OR “prediction model” OR “risk prediction” OR “risk assessment” OR “risk evaluation” OR “risk score” OR “risk stratification model” OR “prediction tool*” OR “prognostic model” OR “stratification model”).

For the Chinese databases (CNKI, Wanfang, VIP, and CBM), the following Chinese search terms and their combinations were used:

(“脑小血管病” OR “脑微血管病” OR “脑白质病变” OR “腔隙性脑梗死” OR “脑萎缩” OR “脑微出血”).

AND (“认知障碍” OR “认知下降” OR “认知损害” OR “失智症” OR “阿尔兹海默症” OR “痴呆”).

AND (“模型” OR “预测模型” OR “风险预测” OR “预测因子” OR “风险分层” OR “风险评估” OR “危险因素”).

The search strategies were adapted to the syntax and requirements of each database. In addition to peer-reviewed literature, gray literature such as conference proceedings, dissertations, government reports, and institutional documents were also included, where applicable, to ensure the comprehensiveness of the evidence base. Studies conducted in both English and Chinese were considered to minimize language bias.

### 2.3 Study screening and data extraction

The studies retrieved based on the search terms were imported into EndNote. Two researchers independently screened the studies and extracted data according to the inclusion and exclusion criteria. In accordance with the established guidelines for systematic reviews of risk prediction models, the CHARMS (Moons et al., 2014) was used to pre-design the data extraction form. Any discrepancies encountered during the extraction process were discussed and resolved. In cases where consensus could not be reached, a third researcher intervened and made the final decision to ensure agreement among all parties. The extracted data included the first author, year of publication, study design, sample size, incidence rates, year of data collection, outcome events, assessment tools, variable selection, model validation methods, area under the receiver operating characteristic curve (AUC), calibration methods, specificity, sensitivity, accuracy, and other relevant information. For studies reporting AUC values without standard errors (SEs) or confidence intervals (CIs), we contacted the corresponding authors by email up to two times to obtain the missing data. If unavailable, SEs were estimated using established methods based on reported 95% CIs or when sufficient information was provided, using the method of Hanley and McNeil. Studies in which SEs could not be obtained or reliably estimated were excluded from the quantitative synthesis but included in the qualitative analysis.

### 2.4 Study quality assessment

In the systematic review of prediction model studies, the Prediction Model Risk of Bias Assessment Tool (PROBAST; Wolff et al., 2019) was used to evaluate the risk of bias in the constructed prediction models. The risk of bias was assessed across four dimensions: study population, predictor variables, outcome, and data analysis. The applicability of the models was evaluated based on three dimensions: study population, predictor variables, and outcome. If the risk of bias or applicability risk in any dimension was rated as “high” or “unclear,” the overall risk was classified as “high.” The assessment was performed independently by two researchers, and any discrepancies were resolved by a third researcher.

### 2.5 Statistical analysis

Meta-analysis of the predictors was performed using RevMan 5.4 software. The outcome measure was a binary variable, with odds ratios (ORs) and 95% confidence intervals (CIs) as effect indicators. Heterogeneity was assessed using the I^2^ and Q tests. A fixed-effects model was selected if *p* > 0.1 and I^2^ < 50%, indicating low heterogeneity, whereas a random-effects model was chosen if *p* < 0.1 and I^2^ ≥ 50%, indicating high heterogeneity. A *p*-value of < 0.05 was considered statistically significant. Sensitivity analysis was conducted by sequentially excluding studies to evaluate their impact on the pooled effect size and assess the robustness of the results across different effect models. Publication bias was evaluated using Egger’s test, with *p* < 0.05 considered statistically significant. Statistical analysis of the AUC was performed using the R software. If a study reported only the 95% CI of the AUC without a standard error (SE), the corresponding author was contacted to obtain the SE. If this was not possible, the SE was estimated by dividing the 95% CI by 3.92 (Hanley and McNeil, 1982).

## 3 Results

### 3.1 Study retrieval process and findings

**Figure 1:**
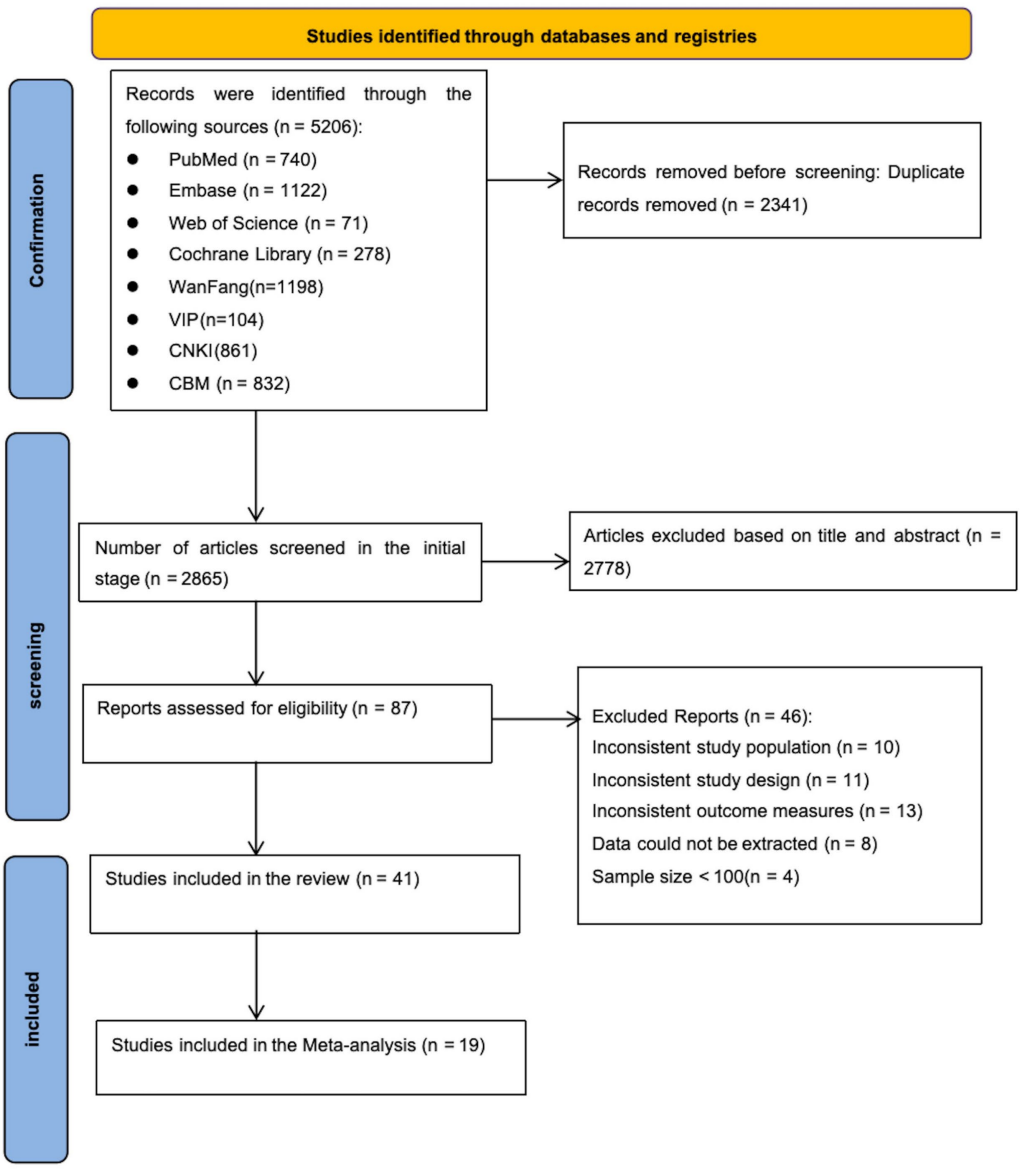
Preferred Reporting Items for Systematic reviews and Meta-Analyses (PRISMA) flowchart of literature search and selection.

Figure 1 shows the flowchart of the study selection process following the Preferred Reporting Items for Systematic Reviews and Meta-Analyses (PRISMA) 2020 guidelines. A total of 5,206 records were initially identified through database searches. After removing duplicates, 2,865 unique records remained. Following title and abstract screening, 87 potentially eligible studies were assessed for full-text review. Of these, 46 studies were excluded for the following reasons: incompatible study populations (*n* = 10), inappropriate study designs (*n* = 11), incompatible outcome measures (*n* = 13), unavailable or unextractable data (*n* = 8), and small sample sizes <100 (*n* = 4). Finally, 19 studies met the inclusion criteria and were included in the meta-analysis. Detailed reasons for exclusion at each stage are shown in Figure 1.

### 3.2 Basic characteristics of study

A total of 19 studies (Dai et al., 2024; Fu et al., 2025; Feng and Li, 2025; Zhang, 2024; Cao et al., 2022; Li et al., 2025; Wang W. et al., 2024; Wang D.M. et al., 2024; Wang et al., 2021; Wang Y. et al., 2024; Qi et al., 2023; Guo et al., 2024; Guo et al., 2021; Hou et al., 2022; Huang et al., 2025; Li et al., 2024; Lin et al., 2025; Zhang L. et al., 2022; Zhu et al., 2024) were included, with the study population primarily consisting of patients with cerebral small vessel disease. In terms of assessment tools, 13 studies (Dai et al., 2024; Fu et al., 2025; Cao et al., 2022; Wang W. et al., 2024; Wang et al., 2021; Wang Y. et al., 2024; Guo et al., 2021; Hou et al., 2022; Huang et al., 2025; Li et al., 2024; Zhang L. et al., 2022; Zhu et al., 2024) used the Montreal Cognitive Assessment (MoCA), 4 studies (Feng and Li, 2025; Zhang, 2024; Guo et al., 2024; Lin et al., 2025) employed the Mini-Mental State Examination (MMSE), and 2 studies (Li et al., 2025; Wang W. et al., 2024) used both MoCA and MMSE. The sample sizes of the included studies ranged from 140 to 415 participants, with the incidence of cognitive impairment varying between 25.16 and 78.57%. All studies were published within the last 5 years. In terms of study design, 18 studies (Dai et al., 2024; Fu et al., 2025; Feng and Li, 2025; Zhang, 2024; Cao et al., 2022; Li et al., 2025; Wang W. et al., 2024; Wang D.M. et al., 2024; Wang et al., 2021; Wang Y. et al., 2024; Qi et al., 2023; Guo et al., 2024; Guo et al., 2021; Hou et al., 2022; Huang et al., 2025; Li et al., 2024; Zhang L. et al., 2022; Zhu et al., 2024) were retrospective, while 1 study (Lin et al., 2025)was a prospective cohort study. Regarding outcome events, 14 studies (Dai et al., 2024; Fu et al., 2025; Feng and Li, 2025; Zhang, 2024; Wang et al., 2021; Wang Y. et al., 2024; Qi et al., 2023; Guo et al., 2024; Guo et al., 2021; Hou et al., 2022; Li et al., 2024; Lin et al., 2025; Zhang L. et al., 2022; Zhu et al., 2024) focused on cognitive impairment, 3 studies (Cao et al., 2022; Li et al., 2025; Zhu et al., 2024) focused on mild cognitive impairment, and 2 studies (Wang W. et al., 2024; Wang D.M. et al., 2024) focused on vascular cognitive impairment. The basic characteristics of the included studies are summarized in Table 2.

**Table 2:** Overview of basic data of the included studies.

Author	Year	Outcome event	Study design	Cognitive impairment assessment tools	Subject	Collection time	Sample size (I/E)	Incidence (I/E) (%)
Dai J (Dai et al., 2024)	2024	CI	Retrospective study	MoCA	CSVD	2020.01–2022.05	173/74	35.26/32.43
Fu H (Fu et al., 2025)	2025	CI	Retrospective study	MoCA	CSVD	2020.04–2024.04	200	60
Feng XM (Feng and Li, 2025)	2025	CI	Retrospective study	MMSE	MLI	2022.01–2024.01	252	78.57
Zhang GX (Zhang, 2024)	2024	CI	Retrospective study	MMSE	LA	2020.06–2023.04	273/117	35.1
Cao Q (Cao et al., 2022)	2022	MCI	Retrospective study	MoCA	CSVD	2019.12–2021.03	226	35.84
Li SX (Li et al., 2025)	2025	MCI	Retrospective study	MoCA+MMSE	CSVD	2019.07–2022.08	312	59.6
Wang W (Wang W. et al., 2024)	2024	VCI	Retrospective study	MoCA+MMSE	CSVD	2018.09–2021.08	159/67	25.16/25.37
Wang DM (Wang D.M. et al., 2024)	2024	VCI	Retrospective study	MoCA	CSVD	2020.01–2020.01	200	40
Wang H (Wang et al., 2021)	2021	CI	Retrospective study	MoCA	CSVD	2019.03–2021.03	200	54
Wang Y (Wang Y. et al., 2024)	2024	CI	Retrospective study	MoCA	CSVD	2016.12–2022.12	415	49.6
Qi LR (Qi et al., 2023)	2023	CI	Retrospective study	MoCA	CSVD	2020.09–2022.05	140/30	64.3/66.7
Guo XM (Guo et al., 2024)	2024	CI	Retrospective study	MMSE	CSVD	2017.01–2022.12	247	33.6
Guo XM (Guo et al., 2021)	2021	CI	Retrospective study	MoCA	CSVD	2019.07–2020.07	275	59.6
Hou L (Hou et al., 2022)	2022	CI	Retrospective study	MoCA	CSVD	2019.09–2021.05	147	44.9
Huang YZ (Huang et al., 2025)	2025	MCI	Retrospective study	MoCA	CSVD	2019.11–2020.8	332	50
Li N (Li et al., 2024)	2024	CI	Retrospective study	MoCA	CSVD	2022.07–2023.10	265/112	68.7/61.6
Lin GH (Lin et al., 2025)	2025	CI	Prospective study	MMSE	CSVD	2019.09–2024.01	165/83	50.9/53
Zhang L (Zhang L. et al., 2022)	2022	CI	Retrospective study	MoCA	CSVD	2019.12–2021.12	159	73
Zhu FF (Zhu et al., 2024)	2024	CI	Retrospective study	MoCA	CSVD	2019.10–2022.10	227/70	44.1/22.9

CI, cognitive impairment; VCL, vascular cognitive impairment; MCI, mild cognitive impairment; MoCA, Montreal Cognitive Assessment; MMSE, Mini-Mental State Examination; CSVD, Cerebral Small Vessel Disease; MLI, Mild Leukoaraiosis; LA, Leukoaraiosis; I, internal validation; E, external validation.

### 3.3 Basic characteristics of the risk prediction model

**Figure 2:**
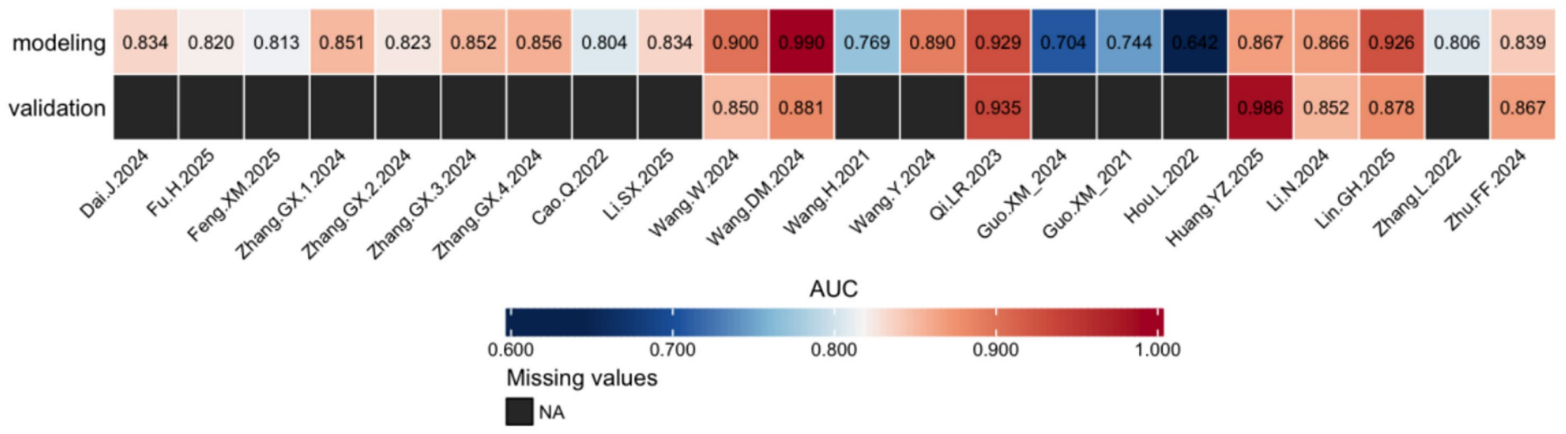
AUC values of the predictive models included in the study.

A total of 19 studies reported 27 risk prediction models. In terms of variable selection, 11 studies (Dai et al., 2024; Cao et al., 2022; Wang W. et al., 2024; Wang D.M. et al., 2024; Qi et al., 2023; Guo et al., 2024; Guo et al., 2021; Hou et al., 2022; Lin et al., 2025; Zhang L. et al., 2022; Zhu et al., 2024) used multivariate analysis for variable selection; 4 studies (Li et al., 2025; Wang W. et al., 2024; Huang et al., 2025; Li et al., 2024) combined multivariate analysis with Least Absolute Shrinkage and Selection Operator (LASSO) regression for variable selection; and another 4 studies (Fu et al., 2025; Feng and Li, 2025; Wang W. et al., 2024) employed a combination of univariate and multivariate analysis for variable selection. Regarding modeling methods, 15 studies (Dai et al., 2024; Fu et al., 2025; Feng and Li, 2025; Li et al., 2025; Wang W. et al., 2024; Wang D.M. et al., 2024; Wang et al., 2021; Wang Y. et al., 2024; Qi et al., 2023; Guo et al., 2024; Guo et al., 2021; Hou et al., 2022; Li et al., 2024; Zhang L. et al., 2022; Zhu et al., 2024)used logistic regression to construct models; four studies (Zhang, 2024; Cao et al., 2022; Huang et al., 2025; Lin et al., 2025) employed machine learning algorithms, constructing seven risk prediction models, including Random Forest, AdaBoost, and Support Vector Machine (SVM). For model calibration evaluation, 5 studies (Dai et al., 2024; Wang W. et al., 2024; Wang et al., 2021; Wang Y. et al., 2024; Huang et al., 2025) evaluated using the Hosmer–Lemeshow goodness-of-fit test, and 7 studies (Dai et al., 2024; Fu et al., 2025; Zhang, 2024; Wang W. et al., 2024; Wang D.M. et al., 2024; Huang et al., 2025; Zhang L. et al., 2022) assessed calibration using calibration curves. In terms of clinical benefit evaluation, three studies (Wang W. et al., 2024; Wang D.M. et al., 2024; Zhang L. et al., 2022) employed the clinical decision curve. Regarding model validation, nine studies (Dai et al., 2024; Fu et al., 2025; Zhang, 2024; Li et al., 2025; Wang W. et al., 2024; Wang D.M. et al., 2024; Li et al., 2024; Zhang L. et al., 2022) performed only internal validation. Only 1 study (Zhu et al., 2024) reported external validation, which included 70 patients with cerebral small vessel disease (mean age 67.71 ± 9.14 years) recruited between October 2019 and September 2020. The incidence of cognitive impairment was 22.86%, and the external validation AUC was 0.867, the remaining nine studies (Feng and Li, 2025; Cao et al., 2022; Wang et al., 2021; Wang Y. et al., 2024; Qi et al., 2023; Guo et al., 2024; Guo et al., 2021; Hou et al., 2022; Huang et al., 2025) lacked validation sets. The AUC values of the included risk prediction models ranged from 0.642 to 0.990 (Figure 2). The detailed characteristics of the models are summarized in Table 3.

**Table 3:** The basic characteristics of the prediction models.

Author	Variable selection	Model development methods	Model performance			Accuracy	Sensitivity	Specificity
			AUC (95% CI) (I/E)	Calibration	Model validation			
Dai J (Dai et al., 2024)	MA	LR, Nomogram	0.834 (0.791 ~ 0.902)	Calibration curves, H-L	Bootstrap	-	-	-
Fu H (Fu et al., 2025)	UA, MA	LR, Nomogram	0.820 (0.758–0.897)/−	Calibration curves	Bootstrap	-	89.7	75
Feng XM (Feng and Li, 2025)	UA, MA	LR	0.813 (0.728 ~ 0.898)	-	-	54.333	77	76
Zhang GX (Zhang, 2024)	UA, MA	LR RF SVM BPNN	0.851 0.823 0.852 0.856	Calibration curves	5-Fold Cross Validation	78.63 76.07 81.02 79.49	-	-
Cao Q (Cao et al., 2022)	MA	RF	0.804	-	-	-	-	-
Li SX (Li et al., 2025)	LASSO, MA	LR, Nomogram	0.834 (0.789 ~ 0.879)	-	Internal validation	-	66.7	88.1
Wang W (Wang W. et al., 2024)	LASSO, MA	LR	0.90 (0.84 ~ 0.96)/0.80 (0.74 ~ 0.96)	Calibration curves, H-L, DCA	Cross-Validation	-	91.52/91.48	73.17/65
Wang DM (Wang D.M. et al., 2024)	MA	LR, Nomogram	0.990 (0.980 ~ 1.000)/0.881 (0.796 ~ 0.967)	Calibration curves	Bootstrap	-	-	-
Wang H (Wang et al., 2021)	UA, MA	LR	0.769	H-L	-	-	55.6	87
Wang Y (Wang Y. et al., 2024)	MA	LR, Nomogram	0.890 (0.859 ~ 0.921)	H-L, DCA	-	-	-	-
Qi LR (Qi et al., 2023)	MA	LR	0.929/0.935	-	-	-	-	-
Guo XM (Guo et al., 2024)	MA	LR	0.704 (0.633 ~ 0.766)	-	-	-	-	-
Guo XM (Guo et al., 2021)	MA	LR, Nomogram	0.744	-	-	-	71.3	69.4
Hou L (Hou et al., 2022)	MA	LR	0.642	-	-	-	69.7	59.3
Huang YZ (Huang et al., 2025)	MA, LASSO	Boruta, Nomogram	0.867/0.986	Calibration curves, H-L	-	-	-	-
Li N (Li et al., 2024)	MA, LASSO	LR	0.866 (0.823–0.909)/0.852 (0.781–0.923)	-	10-fold cross-validation	38.857	75.3/76.9	79.7/74
Lin GH (Lin et al., 2025)	MA	AutoGluon	0.926 (0.875–0.961)	-	Internal validation	88.4/81.93	88.10/86.36	88.89/76.92
Zhang L (Zhang L. et al., 2022)	MA	LR, Nomogram	0.806	Calibration curves, DCA	Bootstrap	-	-	-
Zhu FF (Zhu et al., 2024)	MA	LR, Nomogram	0.839/0.867	-	Internal validation + External validation	-	-	-

UA, univariate analysis; MA, multivariate analysis; LASSO, Least Absolute Shrinkage and Selection Operator; LR, logistic regression; RF, random forest; SVM, support vector machine; BPNN, backpropagation neural network; AUC, area under the receiver operating characteristic curve; H-L, Hosmer–Lemeshow Goodness-of-Fit Test; DCA, decision curve analysis; -, not described.

### 3.4 Risk of bias and applicability assessment

The Prediction model Risk Of Bias ASsessment Tool (PROBAST) was used to systematically evaluate the risk of bias and applicability of the 19 included studies across four domains: participants, predictors, outcomes, and analysis. To ensure the reliability of the assessment, two reviewers independently performed the evaluations, and any discrepancies were resolved through discussion or adjudication by a third reviewer. Inter-rater reliability was quantified using Cohen’s kappa statistic, interpreted according to the Landis–Koch criteria: values >0.80 indicate “almost perfect agreement,” 0.60–0.80 “substantial agreement,” 0.40–0.60 “moderate agreement,” and <0.40 “poor agreement.” In this study, *κ* = 0.78, demonstrating substantial agreement and good inter-rater reliability. The summarized results of the PROBAST assessment are presented in Table 4.

**Table 4:** Bias risk and applicability assessment of the included studies.

Study	ROB				Applicability			Overall	
	Participants	Predictors	Outcome	Analysis	Participants	Predictors	Outcome	ROB	Applicability
Dai J (Dai et al., 2024)	+	?	+	+	−	−	+	+	−
Fu H (Fu et al., 2025)	+	?	?	+	−	−	+	+	−
Feng XM (Feng and Li, 2025)	+	?	?	+	−	−	+	+	−
Zhang GX (Zhang, 2024)	+	?	?	−	−	−	+	+	−
Cao Q (Cao et al., 2022)	+	?	?	+	−	−	+	+	−
Li SX (Li et al., 2025)	+	+	+	+	−	−	+	+	−
Wang W (Wang W. et al., 2024)	+	?	+	+	+	−	+	+	+
Wang DM (Wang D.M. et al., 2024)	+	?	?	+	−	−	+	+	−
Wang H (Wang et al., 2021)	+	?	?	+	−	−	+	+	−
Wang Y (Wang Y. et al., 2024)	+	?	?	+	−	−	+	+	−
Qi LR (Qi et al., 2023)	+	?	?	+	−	−	+	+	−
Guo XM (Guo et al., 2024)	+	?	?	+	−	+	+	+	+
Guo XM (Guo et al., 2021)	+	?	?	+	−	+	+	+	+
Hou L (Hou et al., 2022)	+	?	?	+	−	+	+	+	+
Huang YZ (Huang et al., 2025)	+	?	?	−	−	−	+	+	−
Li N (Li et al., 2024)	+	?	?	+	+	−	+	+	+
Lin GH (Lin et al., 2025)	−	?	?	+	−	−	+	+	−
Zhang L (Zhang L. et al., 2022)	+	?	?	+	−	−	+	+	−
Zhu FF (Zhu et al., 2024)	+	?	?	+	−	−	+	+	−

− = Low bias risk, high applicability; + = High bias risk, low applicability.

#### 3.4.1 Domain of participants

Among all the studies, only one (Lin et al., 2025); was prospective, with a low risk of bias. The remaining 18 studies (Dai et al., 2024; Fu et al., 2025; Feng and Li, 2025; Zhang, 2024; Cao et al., 2022; Li et al., 2025; Wang W. et al., 2024; Wang D.M. et al., 2024; Wang et al., 2021; Wang Y. et al., 2024; Qi et al., 2023; Guo et al., 2024; Guo et al., 2021; Hou et al., 2022; Huang et al., 2025; Li et al., 2024; Zhang L. et al., 2022; Zhu et al., 2024) were retrospective, with a high risk of bias.

#### 3.4.2 Domain of predictors

One study (Li et al., 2025) used multi-center data collection, which may introduce variations in data collection methods, leading to a higher risk of bias. The bias risk for the other studies (Dai et al., 2024; Fu et al., 2025; Feng and Li, 2025; Zhang, 2024; Cao et al., 2022; Wang W. et al., 2024; Wang D.M. et al., 2024; Wang et al., 2021; Wang Y. et al., 2024; Qi et al., 2023; Guo et al., 2024; Guo et al., 2021; Hou et al., 2022; Huang et al., 2025; Li et al., 2024; Lin et al., 2025; Zhang L. et al., 2022; Zhu et al., 2024) is unclear, primarily because it was not explicitly stated whether predictive factors were assessed in the absence of outcome data.

#### 3.4.3 Domain of outcomes

Three studies (Dai et al., 2024; Li et al., 2025; Wang et al., 2024) were determined to have a high risk of bias because of failure to exclude confounding factors in the outcome definitions. The remaining studies in this domain had an “unclear” risk of bias, mainly for two reasons: first, it was not stated whether the time interval between outcome determination and predictive factor assessment was appropriate; second, it was not reported whether blinding was applied when determining the outcomes (i.e., whether the outcome assessors were aware of the predictive factors).

#### 3.4.4 Domain of analysis

With the exception of two studies (Zhang, 2024; Huang et al., 2025) assessed as having a low bias risk, the other 17 studies (Dai et al., 2024; Fu et al., 2025; Feng and Li, 2025; Cao et al., 2022; Li et al., 2025; Wang W. et al., 2024; Wang D.M. et al., 2024; Wang et al., 2021; Wang Y. et al., 2024; Qi et al., 2023; Guo et al., 2024; Guo et al., 2021; Hou et al., 2022; Li et al., 2024; Lin et al., 2025; Zhang L. et al., 2022; Zhu et al., 2024) were considered high risk. Among them, 4 studies (Dai et al., 2024; Fu et al., 2025; Cao et al., 2022; Wang W. et al., 2024) had an increased risk of bias due to a low event-to-predictor ratio (EPV < 20), and the remaining studies (Feng and Li, 2025; Li et al., 2025; Wang W. et al., 2024; Wang et al., 2021; Wang Y. et al., 2024; Qi et al., 2023; Guo et al., 2024; Guo et al., 2021; Hou et al., 2022; Li et al., 2024; Lin et al., 2025; Zhang L. et al., 2022; Zhu et al., 2024) did not report how missing data were handled.

#### 3.4.5 Applicability assessment

Five studies (Wang W. et al., 2024; Guo et al., 2024; Guo et al., 2021; Hou et al., 2022; Li et al., 2024) had poor applicability. Two studies (Wang W. et al., 2024; Li et al., 2024) focused exclusively on elderly populations, while the other three (Guo et al., 2024; Guo et al., 2021; Hou et al., 2022) focused on models predicting the risk of cognitive impairment based on specific factors such as blood eGFR (estimated glomerular filtration rate) and NLR (neutrophil-to-lymphocyte ratio), insulin resistance, and neutrophils. The applicability of the remaining 14 studies (Dai et al., 2024; Fu et al., 2025; Feng and Li, 2025; Zhang, 2024; Cao et al., 2022; Li et al., 2025; Wang W. et al., 2024; Wang et al., 2021; Wang Y. et al., 2024; Qi et al., 2023; Huang et al., 2025; Lin et al., 2025; Zhang L. et al., 2022; Zhu et al., 2024) was considered good.

**Figure 3:**
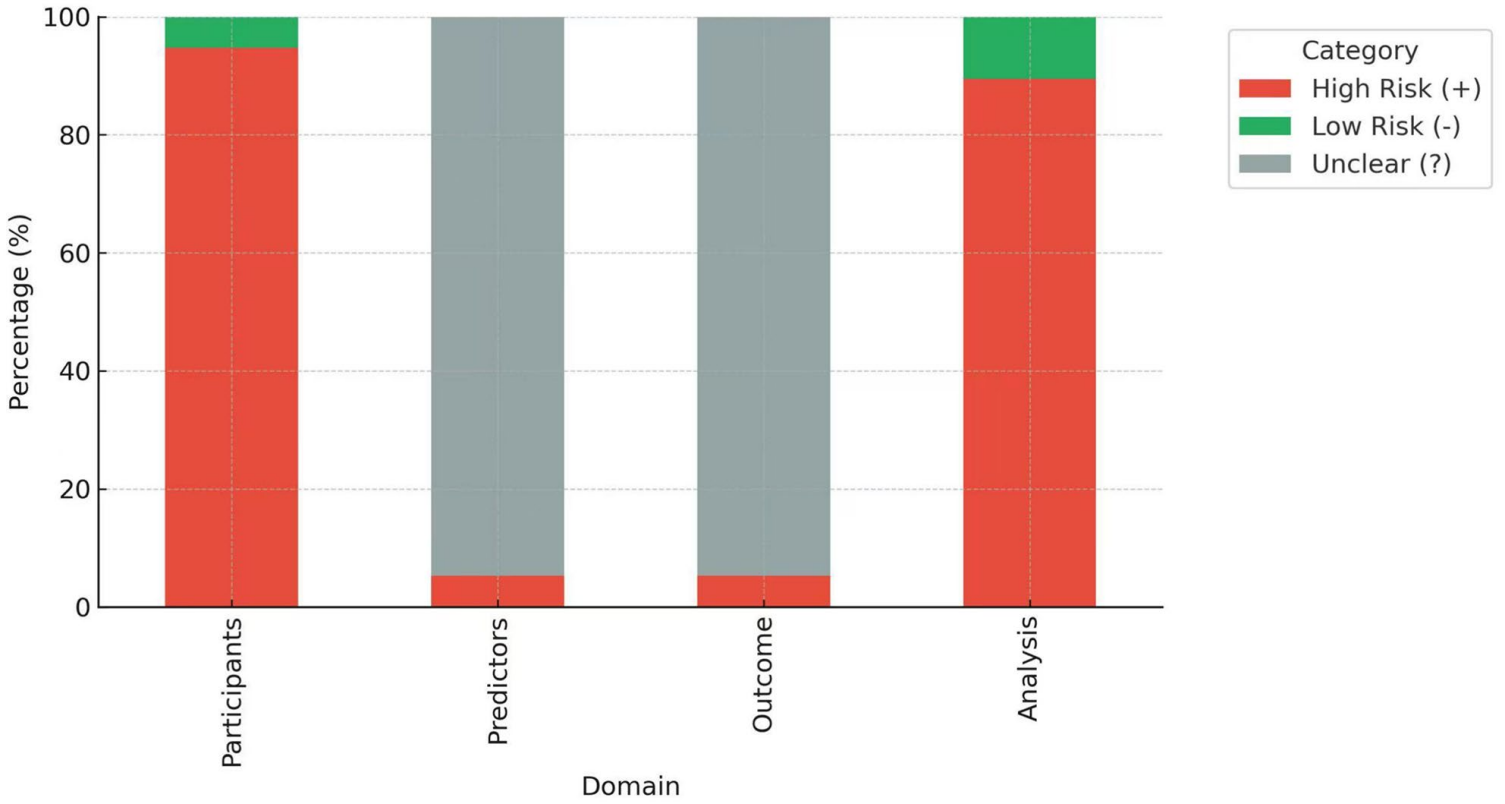
Distribution of PROBAST risk of bias.

In summary, the risk of bias in the domains of participants (94.7%) and analysis (89.5%) was found to be high, while the risk of bias in the domains of predictors (94.7%) and outcomes (94.7%) was primarily rated as “uncertain,” which may be due to insufficient information in the original study reports. The quantified results of the PROBAST risk of bias assessment are presented in Table 5 and Figure 3.

**Table 5:** Quantitative results of PROBAST risk of bias assessment.

Domain	High risk (+)	Low risk (−)	Unclear (?)
Participants	18 (94.7%)	1 (5.3%)	0 (0%)
Predictors	1 (5.3%)	0 (0%)	18 (94.7%)
Outcome	1 (5.3%)	0 (0%)	18 (94.7%)
Analysis	17 (89.5%)	2 (10.5%)	0 (0%)

### 3.5 Meta-analysis results

**Figure 4:**
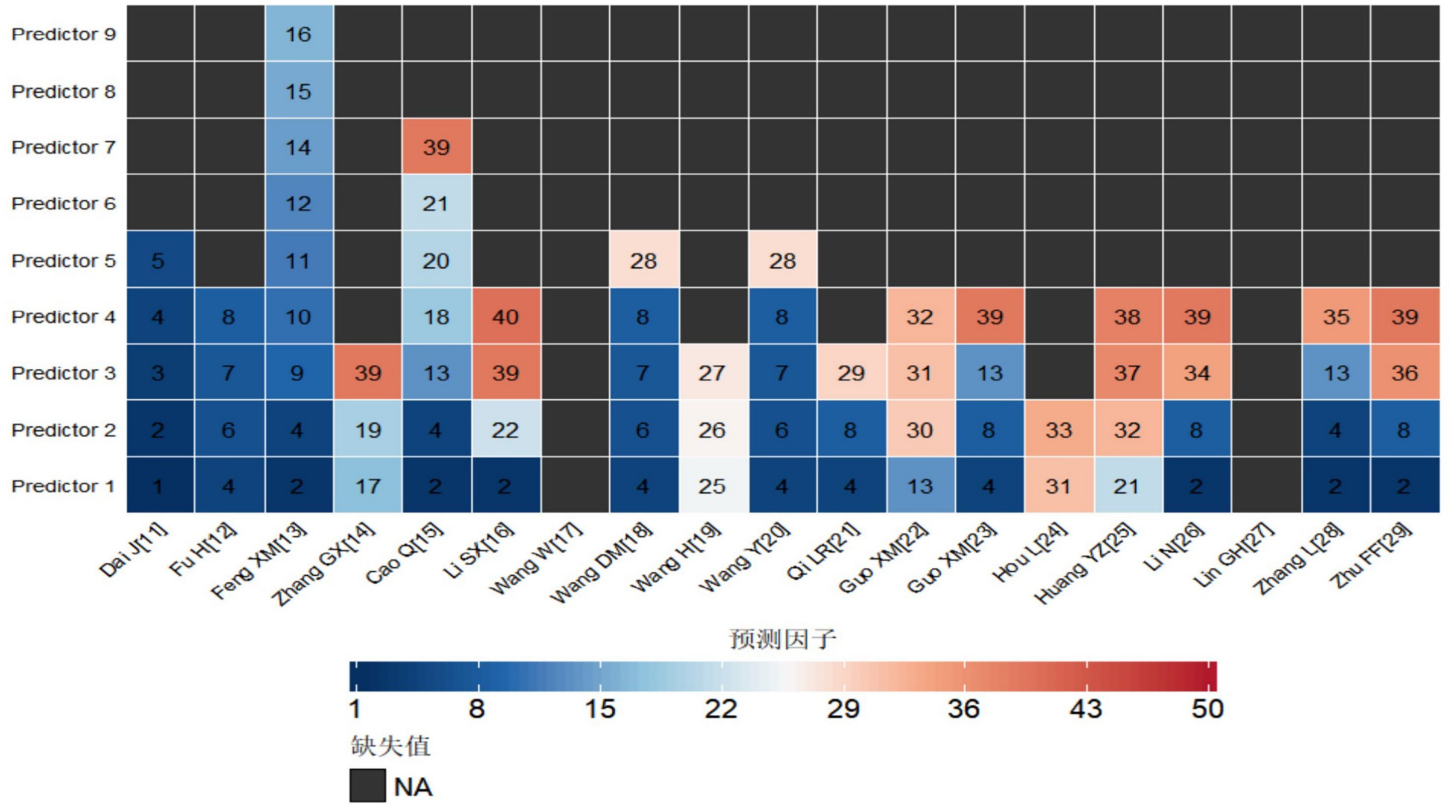
Predictors included in the prediction. 1, C-reactive protein (CRP); 2, Hypertension (HTN); 3, Carotid atherosclerosis; 4, Hyperhomocysteinemia (HHcy); 5, Degree of cerebral white matter lesions (WML); 6, Cystatin C (CysC); 7, Triglyceride-glucose index (TyG index); 8, High-load cerebral small vessel disease (CSVD); 9, Alcohol consumption; 10, Exercise duration <15 h; 11, Family history of dementia; 12, National Institutes of Health Stroke Scale score (NIHSS score); 13, Educational level; 14, Frontal lobe infarction; 15, Thalamic infarction; 16, Basal ganglia infarction; 17, Assessment of intracranial arterial stenosis; 18, Sleep disorder (SD); 19, Severity grading and location of leukoaraiosis (LA); 20, Living alone; 21, Diabetes mellitus (DM); 22, Number of lacunar infarcts ≥1; 23, Total cholesterol (TC); 24, Low-density lipoprotein cholesterol (LDL-C); 25, Sum of lacunar infarction and lacunar lesion counts (LI); 26, Fazekas scale (Fazekas 评分); 27, Giant cell arteritis score (GCA); 28, Apolipoprotein A (ApoA); 29, Lipoprotein-associated phospholipase A2 (LP-PLA2); 30, Estimated glomerular filtration rate (eGFR); 31, Neutrophil-to-lymphocyte ratio (NLR); 32, Male gender; 33, Fasting blood glucose (FBG); 34, Apolipoprotein A1 (ApoA1); 35, Total score of cerebral small vessel disease on MRI (CSVD MRI total score); 36, Lacunar infarction score; 37, Insulin-like growth factor 1 (IGF-1); 38, White matter lesions (WML); 39, Age; 40, High-density lipoprotein cholesterol (HDL-C).

The 19 studies included 40 predictive factors (Figure 4). A meta-analysis was conducted on the predictive factors reported in at least two studies, including hypertension, homocysteine (Hcy), high burden of CSVD, age, educational level, diabetes, cystatin C, and the TyG index. Due to the inclusion of four studies (Zhang, 2024; Huang et al., 2025; Li et al., 2024; Lin et al., 2025) employing machine learning models, which did not provide the necessary data, these studies were excluded from the meta-analysis. For predictive factors with substantial heterogeneity, a sensitivity analysis was performed, which revealed that the combined results remained stable, demonstrating the robustness of the meta-analysis findings. The analysis indicated that hypertension, Hcy, high burden of CSVD, age, educational level, diabetes, and the TyG index are independent risk factors for cognitive impairment in patients with cerebrovascular disease (*p* < 0.05). The detailed results are presented in Table 6.

**Table 6:** Meta-analysis results of predictors for cognitive impairment in patients with cerebral small vessel disease.

Predictive Factors	Study	Meta-analysis			Model	Heterogeneity	
		OR (95%CI)	*Z*	*p*		I^2^ (%)	*p*
Hypertension	7 (Dai et al., 2024; Feng and Li, 2025; Cao et al., 2022; Li et al., 2025; Li et al., 2024; Zhang L. et al., 2022; Zhu et al., 2024)	2.18 (1.67, 2.83)	5.78	*p* < 0.001	Fixed	0	0.81
Hcy	9 (Dai et al., 2024; Fu et al., 2025; Feng and Li, 2025; Cao et al., 2022; Wang W. et al., 2024; Qi et al., 2023; Guo et al., 2024; Guo et al., 2021)	1.27 (1.11, 1.45)	3.56	*p* < 0.001	Random	88	*p* < 0.001
High CSVD burden	6 (Fu et al., 2025; Wang W. et al., 2024; Qi et al., 2023; Guo et al., 2024; Li et al., 2024; Zhu et al., 2024)	3.51 (1.80, 6.82)	3.70	*p* < 0.001	Random	88	*p* < 0.001
Age	7 (Zhang, 2024; Cao et al., 2022; Li et al., 2025; Wang W. et al., 2024; Wang D.M. et al., 2024; Guo et al., 2021; Li et al., 2024; Zhu et al., 2024)	1.18 (1.08, 1.28)	3.74	*p* < 0.001	Random	90	*p* < 0.001
Educational level	5 (Cao et al., 2022; Wang W. et al., 2024; Guo et al., 2024; Guo et al., 2021; Zhang L. et al., 2022)	0.83 (0.76, 0.91)	4.03	*p* < 0.001	Random	86	*p* < 0.001
Diabetes	2 (Cao et al., 2022; Huang et al., 2025)	4.89 (2.84, 8.44)	5.71	*p* < 0.001	Random	61	0.11
Cystatin C	2 (Fu et al., 2025; Wang et al., 2021)	1.73 (0.54, 5.53)	0.92	0.36	Random	76	0.04
TyG index	2 (Fu et al., 2025; Wang et al., 2021)	7.36 (3.94, 13.76)	6.26	*p* < 0.001	Fixed	0	0.83

### 3.6 AUC value analysis results

**Figure 5:**
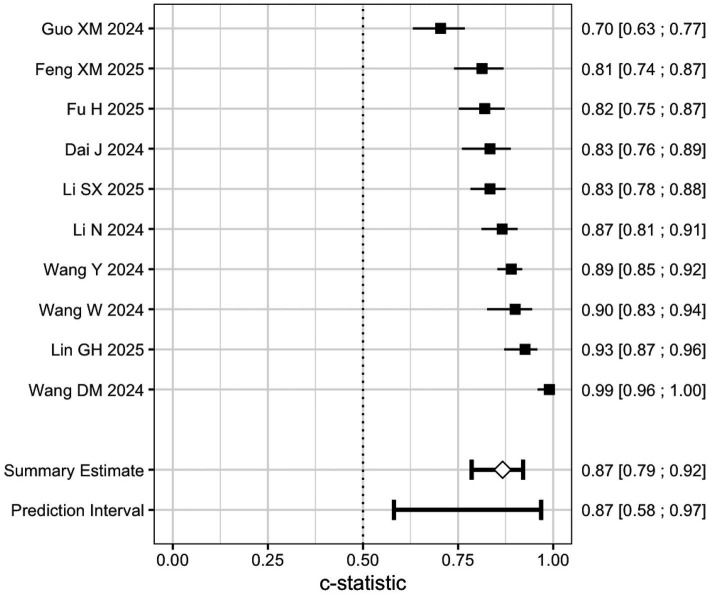
Forest plot of AUC values for the modeling group.

**Figure 6:**
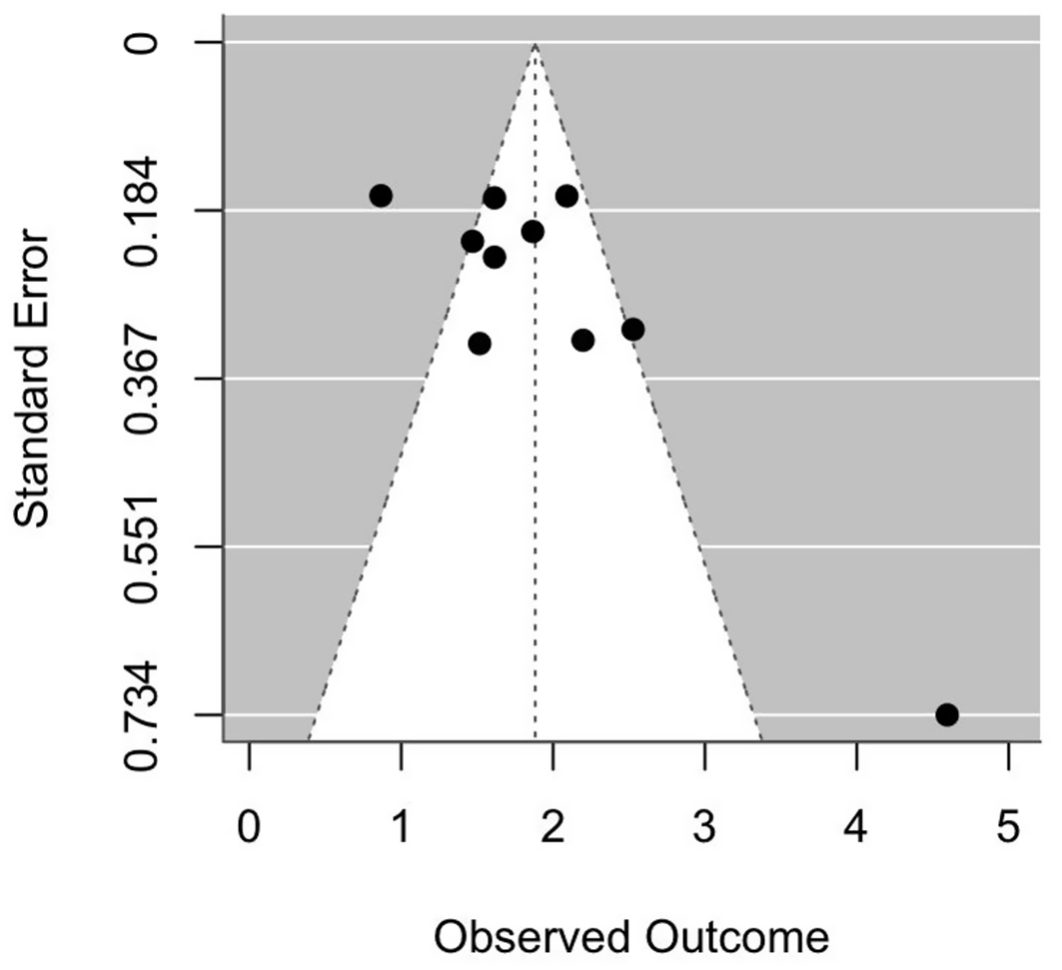
Forest plot of AUC values for the modeling group.

Of the 19 studies included, 9 (Zhang, 2024; Cao et al., 2022; Wang et al., 2021; Qi et al., 2023; Guo et al., 2021; Huang et al., 2025; Zhang L. et al., 2022; Zhu et al., 2024) reported only the AUC values of the prediction models’ test sets, without providing the 95% confidence intervals (CIs) for these AUC values. The remaining 10 studies (Dai et al., 2024; Fu et al., 2025; Feng and Li, 2025; Li et al., 2025; Wang W. et al., 2024; Wang D.M. et al., 2024; Wang Y. et al., 2024; Guo et al., 2024; Li et al., 2024; Lin et al., 2025) reported both AUC values and their corresponding 95% CIs. Statistical analysis of the AUC values and their 95% CIs from the modeling group was performed using R software, and the results are shown in Figure 5. The analysis revealed significant heterogeneity in the modeling group (I^2^ = 88.25%, *p* < 0.0001). Therefore, a random-effects model was applied to pool the effect sizes, yielding a pooled AUC value of 0.87 (95% CI: 0.79–0.92), suggesting moderate predictive accuracy for cognitive impairment. However, due to the large heterogeneity, we conducted a subgroup analysis to explore potential sources of heterogeneity among the included studies. Subgroup analyses were performed based on sample size, number of predictors (<5 or ≥5 predictors), assessment tools, and the incidence of cognitive impairment. The subgroup analysis results indicated that the sample size, number of predictors, and assessment tools could all partially explain the overall heterogeneity. Specifically, studies with smaller sample sizes (<200) and models incorporating five or more predictors showed a higher predictive performance (higher AUC), which contributed to the observed variability in the overall effect size. Differences in predictive performance were also significant across different assessment tools, with studies using the MoCA showing the highest AUC, indicating that the choice of assessment tool was an important source of heterogeneity. In contrast, stratification by the incidence of cognitive impairment showed no significant difference in AUC, suggesting that incidence did not contribute substantially to the overall heterogeneity (as shown in Table 7). Additionally, a funnel plot was generated to assess publication bias. The symmetrical distribution observed on both sides of the funnel plot indicated no significant publication bias (Figure 6).

**Table 7:** Subgroup analysis of AUC results.

Subgroup	Number of studies	I^2^ value (%)	Effect model	AUC (95%CI)	*p*-value
Sample size					
Sample size <200	3	38.4	Fixed	0.900 (0.855 ~ 0.945)	*p* < 0.001
Sample size ≥200	7	96.8	Random	0.848 (0.771 ~ 0.926)	*p* < 0.001
Number of predictors					
<5 predictors	6	85.5	Random	0.844 (0.788 ~ 0.901)	*p* < 0.001
≥5 predictors	4	95	Random	0.890 (0.806 ~ 0.973)	*p* < 0.001
Assessment tools					
MoCA	5	95.5	Random	0.855 (0.810 ~ 0.960)	*p* < 0.001
MMSE	3	93.6	Random	0.816 (0.672 ~ 0.960)	*p* < 0.001
MoCA and MMSE	2	66.4	Random	0.864 (0.800 ~ 0.928)	*p* < 0.001
Incidence					
<50%	6	96.7	Random	0.862 (0.779 ~ 0.945)	*p* < 0.001
≥50%	4	70	Random	0.864 (0.812 ~ 0.915)	*p* < 0.001

## 4 Discussion

Cognitive impairment is highly prevalent in patients with CSVD and has become a major focus of clinical attention (Chojdak-Łukasiewicz et al., 2021). Currently, risk prediction models for cognitive impairment are being actively developed. These models can help healthcare professionals identify high-risk patients early, enabling the implementation of effective preventive measures to reduce the burden of cognitive impairment. However, despite the increasing number of meta-analyses on the risk factors for cognitive impairment, research specifically focused on predictive models for cognitive impairment in the context of CSVD remains limited. This study aims to assess the effectiveness of existing prediction models for CSVD patients through meta-analysis and to provide new insights and directions for further refinement of these models.

### 4.1 The research on predictive models is still in its developmental stage and requires optimization

This study systematically searched both domestic and international databases and ultimately included 19 studies involving 27 prediction models. The AUC values of these models ranged from 0.642 to 0.990, with the majority of models (16/19) having an AUC value ≥0.8, indicating good discriminative ability and predictive performance. However, the PROBAST tool assessment revealed that the majority of the studies exhibited a high risk of bias, particularly regarding participant selection and statistical analysis methods. Specifically, the majority of studies relied on retrospective data, often sourced from a single database and typically collected after the outcome had occurred. This approach may lead to data incompleteness and recording bias, which can undermine the representativeness of the study population and complicate the establishment of a clear causal relationship between predictors and outcomes (Wolff et al., 2019). Therefore, future research should prioritize the use of prospective cohort studies or nested case–control studies to enhance the data reliability and accuracy of causal inferences. In terms of statistical analysis, the risk of bias mainly arises from insufficient event numbers, unclear determination of predictor variables, improper data handling, lack of detailed data processing protocols, and the absence of a comprehensive evaluation of prediction model performance. To address these issues, future model development should prioritize the following aspects: (Markus and de Leeuw, 2023). Strict adherence to the PROBAST tool’s recommendations, ensuring that the number of events per variable (EPV) is ≥20 (Chen et al., 2020), thereby minimizing the potential risk of bias. If increasing the sample size is not feasible, statistical techniques such as regularized regression should be considered to mitigate bias and enhance the model’s stability and reliability. (2) It is essential to avoid selecting predictors solely based on univariate analysis, as this approach may exclude important variables and introduce bias. Instead, more comprehensive and precise variable selection methods, such as multivariable regression analysis or LASSO regression, should be employed (Ranalli et al., 2023). (3) For missing data, it is recommended to employ methods such as listwise deletion, maximum likelihood estimation, and multiple imputation, with detailed descriptions provided in the text, to reduce the risk of overfitting and bias (Ranalli et al., 2023; Sterne et al., 2009) and enhance the validity of the results. However, among the included studies, only two (Zhang, 2024; Huang et al., 2025) employed these methods. (4) The performance evaluation of predictive models should encompass multiple dimensions, with discrimination and calibration being key metrics. In previous studies, the AUC was frequently used to assess the model’s discriminatory power, specifically its ability to distinguish between different outcomes. For model calibration, researchers typically rely on methods such as calibration curves and the Hosmer–Lemeshow (H–L) goodness-of-fit test to evaluate the alignment between predicted values and actual observations. In this study, five articles (Dai et al., 2024; Wang W. et al., 2024; Wang et al., 2021; Wang Y. et al., 2024; Huang et al., 2025) relied exclusively on the Hosmer–Lemeshow (H–L) test for assessing model calibration. However, the *p*-value of the H-L test can be influenced by the sample size and number of groups, which may lead to biased results and fail to fully capture the model’s calibration performance. To ensure a more robust evaluation, it is recommended to incorporate additional methods, such as the visualization of calibration curves and statistical metrics (e.g., the Brier score). Furthermore, the Transparent Reporting of a Multivariable Prediction Model for Individual Prognosis (TRIPOD) guidelines (Collins et al., 2015) should be followed as a reference for the development, validation, and reporting of future risk prediction models.

### 4.2 Analysis of predictors

When constructing predictive models, both simplicity and accuracy are equally important. Increasing the number of predictors or introducing more complex variables does not necessarily enhance model performance; instead, it may increase the risk of overfitting and limit the applicability of the model across different clinical settings. In this study, through meta-analysis, we identified several key predictive factors for cognitive impairment in patients with CSVD, including hypertension, education level, homocysteine (Hcy), high CSVD burden, age, diabetes, and the TyG index.

Research indicates (Wardlaw et al., 2019) that chronic hypertension can impair the structure and function of cerebral small vessels, leading to inadequate brain perfusion, disruption of the blood–brain barrier, and dysfunction of the neurovascular unit. These changes trigger oxidative stress, inflammation, and myelin damage, ultimately accelerating cognitive decline. Individuals with higher education levels generally possess more knowledge and information in the brain, which may enhance the brain’s compensatory ability to cope with aging by altering its structure, biochemical metabolism, and multisynaptic connections, thereby mitigating cognitive impairment (Tessaro et al., 2020). The relationship between Hcy and cognitive impairment remains unclear, but the underlying mechanisms are believed to be complex and multifactorial. Existing meta-analyses have shown (Zhou and Chen, 2019) that plasma total Hcy levels are causally related to the risk of Alzheimer’s disease, with a 15% increase in the risk of Alzheimer’s disease for every 5 μmol/L increase in Hcy levels (Hu et al., 2016). Elevated Hcy levels may lead to cognitive impairment by inducing oxidative stress, accelerating atherosclerosis, damaging the vascular walls, and increasing thrombosis formation. Age is a well-established independent risk factor for cognitive impairment. As individuals age, neurons in the brain gradually atrophy or sustain damage, and CSVD may accelerate these degenerative changes, further impairing memory, attention, and executive function (Fu et al., 2020). CSVD burden serves as a key indicator of the severity of small vessel damage. An elevated burden signifies significant damage to both the structure and function of cerebral vessels, and these pathological changes may accelerate the progression of cognitive decline (Zhang X.Q. et al., 2022). Additionally, this study found that diabetes and the TyG index, as metabolic indicators, are strongly associated with cerebrovascular lesions and cognitive decline, consistent with existing studies (Sun et al., 2020; Wang et al., 2023). These findings underscore the critical role of blood glucose control and insulin sensitivity in maintaining cerebrovascular health. However, only two studies have specifically investigated these factors. Given their potential clinical implications, more high-quality large-scale studies are needed to validate the role of these metabolic indicators in cerebral small vessel disease and cognitive impairment. Furthermore, future research should explore their interactions with other risk factors to establish a more reliable foundation for early screening and intervention.

### 4.3 Limitations

This study included only Chinese- and English-language studies on risk prediction models for cognitive impairment in patients with cerebrovascular disease, which may have resulted in the exclusion of relevant studies published in other languages. As several predictors were reported in only a single study, a meta-analysis was not feasible, which may have introduced bias into the results. Additionally, the majority of the included studies were conducted in Asia, which may have limited the generalizability and applicability of the findings. The sample sizes of the included studies varied substantially (ranging from 140 to 415 participants), which may have affected the stability and robustness of the model performance estimates. Furthermore, most of the prediction models lacked external validation, thereby limiting their generalizability to other populations and clinical settings. Finally, this review included only studies published within the past 5 years to reflect recent advances in prediction modeling and clinical practice. However, this inclusion criterion may have resulted in the omission of well-established classic models that may still be clinically relevant.

## 5 Conclusion

In recent years, with the growing research on the relationship between CSVD and cognitive impairment, the number of prediction models for cognitive impairment in patients with cerebrovascular disease has gradually increased. These models generally exhibit good discriminatory ability, offering the capacity to predict the risk of cognitive impairment, particularly with potential utility for early intervention. However, despite the development of various predictive methods, the majority of studies lack external validation, and the generalizability of these models remains a challenge. For example, some studies rely on data from a single region or a homogeneous sample population, which may limit the generalizability of the model to diverse clinical settings across different populations and regions. Current prediction models typically combine traditional statistical methods (such as regression analysis) with modern machine learning techniques, the latter of which excels in handling large-scale, multidimensional data (e.g., imaging and genomic data). However, these models often face challenges such as small sample sizes, incomplete feature selection, and a lack of external validation, all of which hinder their broader implementation in clinical practice. Moving forward, risk prediction models for cognitive impairment in CSVD must be improved in several key areas. On the one hand, further investigation is required into the application of tree models, support vector machines (SVMs), and artificial intelligence (AI) approaches, including neural network-based machine learning techniques, to improve the accuracy and robustness of prediction models. However, as data diversity expands (e.g., genomics and imaging data), future models should integrate multiple data sources, providing a more comprehensive basis for individualized prediction. This approach would not only enhance the generalizability of prediction models but also aid healthcare professionals in the early identification of high-risk patients and the development of personalized treatment plans, ultimately helping to slow the onset and progression of cognitive impairment.

## Data Availability

The original contributions presented in the study are included in the article/supplementary material, further inquiries can be directed to the corresponding author.

## References

[ref1] Cao Q. Su M. X. Wei J. H. Tang Z. H. Chen B. (2022). A diagnostic model for cerebral small vessel disease with mild cognitive impairment based on random forest algorithm. J. Psychiatry 35, 365–369. doi: 10.3969/j.issn.2095-9346.2022.04.013

[ref2] Chen X. P. Zhang Y. Zhuang Y. Y. Zhang Z. H. (2020). PROBAST: a tool for assessing the risk of bias in diagnostic or prognostic multivariable prediction model studies. Chin. J. Evid. Based Med. 20, 737–744. doi: 10.7507/1672-2531.202005073

[ref3] Chojdak-Łukasiewicz J. Dziadkowiak E. Zimny A. Paradowski B. (2021). Cerebral small vessel disease: a review. Adv. Clin. Exp. Med. 30, 349–356. doi: 10.17219/acem/131216, 33768739

[ref4] Collins G. S. Reitsma J. B. Altman D. G. Moons K. G. M. (2015). Transparent reporting of a multivariable prediction model for individual prognosis or diagnosis (TRIPOD): the TRIPOD statement. BMJ 350:g7594. doi: 10.1136/bmj.g7594, 25569120

[ref5] Dai J. Zhang S. X. Zhao S. S. Zhang X. F. (2024). Analysis of influencing factors and construction and validation of a nomogram model for cognitive impairment in patients with cerebral small vessel disease. Chin. J. Stroke. 19, 1136–1142. doi: 10.3969/j.issn.1673-5765.2024.10.011

[ref6] Das A. S. Regenhardt R. W. Vernooij M. W. Blacker D. Charidimou A. Viswanathan A. (2019). Asymptomatic cerebral small vessel disease: insights from population-based studies. J. Stroke 21, 121–138. doi: 10.5853/jos.2018.03608, 30991799 PMC6549070

[ref7] Feng X. M. Li Y. (2025). Risk factor analysis and prediction model construction for cognitive impairment in patients with multiple lacunar infarction. J. Mod. Med. 53, 172–178. doi: 10.3969/j.issn.1671-7562.2025.02.003

[ref8] Fu H. Gong L. S. Chen L. Zhang L. Jia H. M. Ji Y. L. (2025). Influencing factors of cognitive impairment in patients with cerebral small vessel disease and construction of a risk prediction model. J. Brain Nerv. Dis. 33, 181–185.

[ref9] Fu J. Liu Q. Du Y. Zhu Y. Sun C. Lin H. . (2020). Age- and sex-specific prevalence and modifiable risk factors of mild cognitive impairment among older adults in China: a population-based observational study. Front. Aging Neurosci. 12:578742. doi: 10.3389/fnagi.2020.578742, 33192471 PMC7662098

[ref10] Guo X. M. Lei X. Y. Liu Z. W. Yuan W. S. Wei A. Q. Zhu N. (2024). Construction and evaluation of a nomogram prediction model for cognitive impairment in patients with cerebral small vessel disease based on blood eGFR and NLR. J. Mod. Lab. Med. 39, 85–91. doi: 10.3969/j.issn.1671-7414.2024.05.016

[ref11] Guo X. M. Zhu Y. Li X. Lu Z. Cao Z. Yi X. . (2021). Increased insulin resistance is associated with vascular cognitive impairment in Chinese patients with cerebral small vessel disease. Psychogeriatrics 21, 342–349. doi: 10.1111/psyg.12675, 33641231

[ref12] Hanley J. A. McNeil B. J. (1982). The meaning and use of the area under a receiver operating characteristic (ROC) curve. Radiology 143, 29–36. doi: 10.1148/radiology.143.1.7063747, 7063747

[ref13] Hou L. Zhang S. Qi D. Jia T. Wang H. Zhang W. . (2022). Correlation between neutrophil/lymphocyte ratio and cognitive impairment in cerebral small vessel disease patients: a retrospective study. Front. Neurol. 13:925218. doi: 10.3389/fneur.2022.925218, 35989913 PMC9391025

[ref14] Hu Q. Teng W. Li J. Hao F. Wang N. (2016). Homocysteine and Alzheimer's disease: evidence for a causal link from Mendelian randomization. J Alzheimer's Dis 52, 747–756. doi: 10.3233/JAD-150977, 27031476

[ref15] Huang Y. Huang W. Ma X. Zhao G. Kang J. Li H. . (2025). Nomogram for predicting mild cognitive impairment in Chinese elder CSVD patients based on Boruta algorithm. Front. Aging Neurosci. 17:1431421. doi: 10.3389/fnagi.2025.1431421, 39963470 PMC11830805

[ref16] Hussenoeder F. S. Conrad I. Roehr S. Fuchs A. Pentzek M. Bickel H. . (2020). Mild cognitive impairment and quality of life in the oldest old: a closer look. Qual. Life Res. 29, 1675–1683. doi: 10.1007/s11136-020-02425-5, 31993915 PMC7253517

[ref17] Iadecola C. Duering M. Hachinski V. Joutel A. Pendlebury S. T. Schneider J. A. . (2019). Vascular cognitive impairment and dementia: JACC scientific expert panel. J. Am. Coll. Cardiol. 73, 3326–3344. doi: 10.1016/j.jacc.2019.04.034, 31248555 PMC6719789

[ref18] Li S. X. Duan J. Y. Jin H. H. Wang Y. F. Song J. L. Li X. S. . (2025). Construction of a prediction model for mild cognitive impairment in cerebral small vessel disease based on TCM syndrome elements. Guide J. Tradit. Chin. Med. Pharm. 31, 95–100. doi: 10.13862/j.cn43-1446/r.2025.03.021

[ref19] Li N. Gao Y. Li L. T. Hu Y. D. Ling L. Jia N. . (2024). Development and validation of a nomogram predictive model for cognitive impairment in cerebral small vessel disease: a comprehensive retrospective analysis. Front. Neurol. 15:1373306. doi: 10.3389/fneur.2024.1373306, 38952470 PMC11215066

[ref20] Lin G. Chen W. Geng Y. Peng B. Liu S. Chen M. . (2025). A multimodal MRI-based machine learning framework for classifying cognitive impairment in cerebral small vessel disease. Sci. Rep. 15:13112. doi: 10.1038/s41598-025-97552-9, 40240809 PMC12003736

[ref21] Markus H. S. de Leeuw F. E. (2023). Cerebral small vessel disease: recent advances and future directions. Int. J. Stroke 18, 4–14. doi: 10.1177/17474930221144911, 36575578 PMC9806465

[ref22] Markus H. S. Joutel A. (2025). The pathogenesis of cerebral small vessel disease and vascular cognitive impairment. Physiol. Rev. 105, 1075–1171. doi: 10.1152/physrev.00028, 39965059 PMC12182829

[ref23] Mok V. Kim J. S. (2015). Prevention and Management of Cerebral Small Vessel Disease. J. Stroke 17, 111–122. doi: 10.5853/jos.2015.17.2.111, 26060798 PMC4460330

[ref24] Moons K. G. de Groot J. A. Bouwmeester W. Vergouwe Y. Mallett S. Altman D. G. . (2014). Critical appraisal and data extraction for systematic reviews of prediction modelling studies: the CHARMS checklist. PLoS Med. 11:e1001744. doi: 10.1371/journal.pmed.1001744, 25314315 PMC4196729

[ref25] Qi L. R. Geng X. Zeng R. Hu F. Y. (2023). Risk prediction of cognitive impairment by combining total cerebral small vessel disease imaging burden score with blood inflammatory and metabolic indicators. Neural Injury Neural Regen. 18, 456–460. doi: 10.16780/j.cnki.sjssgncj.20220484

[ref26] Ranalli M. G. Salvati N. Petrella L. Pantalone F. (2023). M-quantile regression shrinkage and selection via the Lasso and elastic net to assess the effect of meteorology and traffic on air quality. Biom. J. 65:e2100355. doi: 10.1002/bimj.202100355, 37743255

[ref27] Salvadori E. Brambilla M. Maestri G. Nicotra A. Cova I. Pomati S. . (2023). The clinical profile of cerebral small vessel disease: toward an evidence-based identification of cognitive markers. Alzheimers Dement. 19, 244–260. doi: 10.1002/alz.12650, 35362229 PMC10084195

[ref28] Sterne J. A. White I. R. Carlin J. B. Spratt M. Royston P. Kenward M. G. . (2009). Multiple imputation for missing data in epidemiological and clinical research: potential and pitfalls. BMJ 338:b2393. doi: 10.1136/bmj.b2393 19564179 PMC2714692

[ref29] Sun Y. Ma C. Sun H. Wang H. Peng W. Zhou Z. . (2020). Metabolism: a novel shared link between diabetes mellitus and Alzheimer's disease. J. Diabetes Res. 2020:4981814. doi: 10.1155/2020/4981814, 32083135 PMC7011481

[ref30] Tessaro B. Hermes-Pereira A. Schilling L. P. Fonseca R. P. Kochhann R. Hübner L. C. (2020). Verbal fluency in Alzheimer's disease and mild cognitive impairment in individuals with low educational level and its relationship with reading and writing habits. Dement Neuropsychol. 14, 300–307. doi: 10.1590/1980-57642020dn14-030011, 32973983 PMC7500813

[ref31] Wang Y. Li Y. Jiao S. S. Ma H. Y. Liu Z. R. (2024). Influencing factors and nomogram model construction for cognitive impairment in patients with cerebral small vessel disease. Pract. J. Cardiac Cerebral Pneumal Vasc. Dis. 32, 57–62. doi: 10.12114/j.issn.1008-5971.2024.00.020

[ref32] Wang H. Ling Q. Wu Y. Zhang M. (2023). Association between the triglyceride glucose index and cognitive impairment and dementia: a meta-analysis. Front. Aging Neurosci. 15:1278730. doi: 10.3389/fnagi.2023.1278730, 38161596 PMC10757637

[ref33] Wang H. Liu M. Chen J. L. Chen L. Fang Z. P. Li Y. (2021). Correlation between cerebral small vessel disease subtypes, extracranial carotid atherosclerosis, and vascular cognitive impairment. J. Chongqing Med. Univ. 46, 1382–1387. doi: 10.13406/j.cnki.cyxb.002910

[ref34] Wang W. Meng Y. K. Zhu S. G. Ge Y. Q. Xu K. (2024). A predictive model of vascular cognitive impairment based on radiomics of white matter hyperintensities in elderly patients with cerebral small vessel disease. Chin. J. Gerontol. 44, 3848–3853. doi: 10.3969/j.issn.1005-9202.2024.16.023

[ref35] Wang D. M. Zou L. J. Bian Y. Feng Q. L. Guo Y. (2024). Characteristics of cognitive impairment, analysis of influencing factors, and construction and validation of a prediction model in patients with cerebral small vessel disease. J. Clin. Intern. Med. 41, 819–822. doi: 10.3969/j.issn.1001-9057.2024.12.011

[ref36] Wardlaw J. M. Smith C. Dichgans M. (2019). Small vessel disease: mechanisms and clinical implications. Lancet Neurol. 18, 684–696. doi: 10.1016/S1474-4422(19)30079-1, 31097385

[ref37] Wolff R. F. Moons K. G. M. Riley R. D. Whiting P. F. Westwood M. Collins G. S. . (2019). PROBAST: a tool to assess the risk of bias and applicability of prediction model studies. Ann. Intern. Med. 170, 51–58. doi: 10.7326/M18-1376, 30596875

[ref38] Zhang G. X. (2024). A comparative study of prediction models for cognitive impairment in patients with leukoaraiosis [dissertation]. Yangzhou: Yangzhou University.

[ref39] Zhang L. Gao F. Zhang Y. Hu P. Yao Y. Zhang Q. . (2022). Analysis of risk factors for the development of cognitive dysfunction in patients with cerebral small vessel disease and the construction of a predictive model. Front. Neurol. 13:944205. doi: 10.3389/fneur.2022.944205, 36034271 PMC9403715

[ref40] Zhang X. Q. Liu S. R. Liu C. X. Fan X. Y. Hou B. You H. . (2022). Study on the correlation between H-type hypertension, total burden of cerebral small vessel disease, and 10-year stroke risk. Pract. J. Cardiac Cerebral Pneumal. Vasc. Dis. 30, 29–34. doi: 10.12114/j.issn.1008-5971.2022.00.313

[ref41] Zhou F. T. Chen S. R. (2019). Hyperhomocysteinemia and risk of incident cognitive outcomes: an updated dose-response meta-analysis of prospective cohort studies. Ageing Res. Rev. 51, 55–66. doi: 10.1016/j.arr.2019.02.006, 30826501

[ref42] Zhu F. Yao J. Feng M. Sun Z. (2024). Establishment and evaluation of a clinical prediction model for cognitive impairment in patients with cerebral small vessel disease. BMC Neurosci. 25:35. doi: 10.1186/s12868-024-00883-y, 39095700 PMC11295716

